# Clinical value of measuring plasma D-dimer levels in patients with esophageal cancer

**DOI:** 10.1186/s13019-024-02895-5

**Published:** 2024-06-22

**Authors:** Hao Chen, Bindong Xu, Qiang Zhang, Pengfei Chen

**Affiliations:** 1 School of Clinical Medicine, Fujian Medical University, No. 88, Jiaotong Road, Fuzhou, Fujian 350004 China; 2 Department of Thoracic and Cardiovascular Surgery, Affiliated Hospital of Putian University, Putian, Fujian China

**Keywords:** Biomarkers, Tumor, D-dimer, Esophageal neoplasms, Fibrin fragment D

## Abstract

**Background:**

Esophageal cancer represents a significant public health concern; however, reliable diagnostic and prognostic markers have not been established. This study aimed to investigate the clinical value of plasma D-dimer levels in patients with esophageal cancer.

**Methods:**

Overall, 120 patients with esophageal cancer who underwent radical surgical resection at our department between January 2019 and 2020 were included (esophageal cancer group). Plasma D-dimer levels were measured preoperatively and on postoperative days 1 and 14. Additionally, 60 healthy participants (control group) with measured plasma D-dimer levels were included. The preoperative D-dimer levels and positive D-dimer test rates were compared between the groups. The 3-year survival rate in patients with esophageal cancer was calculated using the Kaplan–Meier method.

**Results:**

Preoperative D-dimer concentration in the esophageal cancer group was (0.65 ± 0.859 **µ**g/mL) significantly higher than that in the control group (0.32 ± 0.369 **µ**g/mL). The positivity rate in the esophageal cancer group (35.0%, 42/120) was significantly higher than that in the control group (15%, 9/60). D-dimer concentrations were significantly higher 1 day postoperatively than preoperatively. Conversely, D-dimer concentrations were significantly lower 14 days postoperatively than preoperatively. Patients in the esophageal cancer group with plasma D-dimer concentrations ≤ 0.5 µg/mL had significantly higher 3-year survival rates than those with higher concentrations. In the logistic multivariate analysis, tumor pathological stage and preoperative plasma D-dimer levels were independent prognostic factors of 3-year survival rates in patients with esophageal cancer.

**Conclusion:**

Plasma D-dimer concentrations are clinically valuable in esophageal cancer diagnosis, postoperative recurrence monitoring, and prognosis prediction.

## Background

Esophageal cancer is one of the most common malignant tumors of the digestive tract [1]. Approximately 200,000 people worldwide die from esophageal cancer every year, making it the fourth deadliest cancer, with more than half of these deaths occurring in China [2, 3]. The early stages of esophageal cancer lack characteristic clinical manifestations; therefore, most patients seek treatment in the middle or late stages of progression. Surgery remains the primary treatment option [4]. However, despite advancements in surgical techniques, chemotherapy, radiotherapy, and immunotherapy, the 5-year survival rate remains below 30% [5]. Therefore, early detection and prognosis prediction of esophageal cancer are common problems faced by thoracic surgeons. Patients with malignant tumors have hypercoagulable blood, exhibiting abnormalities in the coagulation and fibrinolytic systems, which are related to the formation of thrombi and infiltration of tumor cells [6]. D-dimer is a degradation product of fibrin hydrolyzed by plasmin, and increased D-dimer levels can reflect the physiological hypercoagulable state and hyperfibrinolysis level. Gotta et al. [7] found that plasma D-dimer levels were significantly higher in patients with cancer than in healthy participants. Kharawala et al. [8] found that plasma D-dimer levels in patients with esophageal cancer were related to the number of lymph node metastases. Several clinical reports have examined the relationship between pre-operative D-dimer levels and esophageal cancer stage; moreover, elevated D-dimer levels can be seen in venous thrombotic diseases, pulmonary embolism, infectious diseases, and pregnancy [9]. We aimed to investigate the clinical significance of plasma D-dimer detection in early diagnosis, prognosis prediction, and monitoring of esophageal cancer. One of the limitations is that there may be cases of loss to follow-up.

## Methods

### General information

We selected 120 patients with esophageal cancer (esophageal cancer group) who were admitted to our department for surgical treatment from June 2020 to May 2022, including 70 men and 50 women aged 48–79 years (mean, 65.8 ± 7.10 years).

During the same period, 60 healthy participants (control group) were selected from the physical examination department, including 37 men and 23 women aged 46–88 years (mean, 65.7 ± 7.53 years).

The inclusion criteria for the esophageal cancer group were pathological evidence of malignancy (excluding small cell carcinoma)—established based on electronic gastroscope biopsy—and the absence of prior radiotherapy, chemotherapy, and/or immunotherapy.

The exclusion criteria for this study included preoperative use of aspirin, clopidogrel, low molecular weight heparin, and other drugs that affect coagulation and fibrinolysis; inability to undergo radical surgical resection; esophageal cancer combined with pulmonary embolism, deep vein thrombosis, coronary heart disease, diabetes, liver cirrhosis, cerebral embolism, or other hypercoagulable diseases; and esophageal cancer combined with other malignant tumors.

Among the 120 patients with esophageal cancer, 18 had upper thoracic esophageal cancer, 58 had middle thoracic esophageal cancer, 36 had lower thoracic esophageal cancer, and 8 had cancer of the esophagogastric junction. Upper thoracic and middle thoracic esophageal cancers were treated with thoracic laparoscopy combined with lower three-field esophagectomy. Lower thoracic esophageal and esophagogastric junction cancers were treated with thoracic laparoscopy combined with lower two-field esophagectomy. Following postoperative pathological diagnosis, all esophageal cancers were classified as squamous cell carcinomas, while all esophagogastric junction cancers were classified as adenocarcinomas. The postoperative pathological staging of each tumor was determined according to the 2009 standards of the International Union Against Cancer and the American Joint Committee on Cancer, showing 10 cases of stage IA, 24 of stage IB, 12 of stage IIA, 18 of stage IIB, 20 of stage IIIA, 24 of stage IIIB, and 12 of stage IVA. Among these cases, 38 were classified as well differentiated, 44 as moderately differentiated, and 38 as poorly differentiated.

Patients whose postoperative pathology showed positive lymph node metastasis received chemotherapy. The chemotherapy regimen used for squamous cell carcinoma was the following: paclitaxel 150 mg/m^2^ intravenously infused on day 1 + cisplatin 50 mg/m^2^ intravenously infused on day 1, then every 2 weeks, for a total of 4 times. For adenocarcinoma, the chemotherapy regimen was the following: capecitabine 1000 mg orally bid days 1–14 + oxaliplatin 130 mg/m^2^ intravenously on day 1, then once every 3 weeks, for a total of 4 times.

### D-dimer detection method

Morning blood samples were collected from patients with esophageal cancer prior to the operation and on days 1 and 14 after the operation. A single fasting morning venous blood sample (3 mL) was collected from each healthy participant using a 3-mL vacuum blood collection tube. The anticoagulant reagent 3.8% sodium citrate (100 mL/bottle, Shanghai Enzyme Biotechnology Co., Ltd., No. 5500, Yuanjiang Road, Minhang District, Shanghai) was added to the blood at a ratio of 1:9. Each sample was centrifuged after collection at 168 *g* for 10 min; the temperature during centrifugation was 2–8℃. After the plasma was separated, the test was carried out within 2 h. An automatic latex-enhanced immunoassay was used to detect the plasma D-dimer content in the specimens from the two groups. The normal range of D-dimer concentration is 0–0.5 µg/mL. A positive D-dimer test was defined as having a D-dimer concentration > 0.5 µg/mL.

### Follow-up care

Postoperative follow-up methods for patients with esophageal cancer were implemented as follows: In the first year after surgery, follow-up was conducted in the outpatient clinic every 3 months, and every 6 months thereafter for 2–3 years. Follow-up included peripheral plasma D-dimer measurements, blood cell analysis, a full set of routine biochemistry tests, direct enhancement of neck, chest, liver, gallbladder, and pancreas by computed tomography, and whole-body bone scans.

### Statistical methods

SPSS 18.0 software (IBM Corp., Armonk, New York, USA) was used for data analysis. Quantitative data were subjected to analysis of variance. The Mann–Whitney U test was performed for quantitative data that did not meet the normality standard, while a t-test was performed for quantitative data that met the normality standard. Among the qualitative data, unordered multi-category data were analyzed using the chi-square test. The 3-year survival rate in patients with esophageal cancer was calculated using the Kaplan–Meier method. Related factors affecting the 3-year survival rate in patients with esophageal cancer were analyzed using logistic multivariate analysis. Receiver operating characteristic curves were prepared to determine the accuracy of plasma D-dimer in predicting esophageal cancer outcomes. The area under the curve (AUC) was used to estimate the diagnostic accuracy. Statistical significance was determined at *P* < 0.05.

### Ethics statement

All participants provided informed consent and the study protocol was approved by the hospital ethics committee (approval number: Puyuan Fuyilun [2,023,040]). This study was performed in accordance with the criteria of the Declaration of Helsinki.

## Results

### Comparison of preoperative plasma D-dimer levels in patients with esophageal cancer and the control group

No statistically significant differences were observed in sex and age between the groups (both *P* > 0.05). The preoperative D-dimer concentration** in the esophageal cancer group was (0.65 ± 0.859 **µ**g/mL) significantly higher than that in the control group (0.32 ± 0.369 **µ**g/mL); t = 2.788, **P = 0.003). Additionally, the positivity rate* was also significantly higher in the esophageal cancer group (35.0%) than in the control group (15%; χ^2^ = 6.400, *P = 0.011) (Table 1).

**Table 1:** Comparison of general information and D-dimer levels of the two groups of participants

Group	*n*	Sex		Average age ($\bar x$ ± s)	D-dimer** (µg/mL) ($\bar x$ ± s)	D-dimer* (µg/mL)	
		Male	Female			≤ 0.5 (n)	> 0.5 (n)
Esophageal cancer group	120	74 (61.67)	46 (38.33)	65.7 ± 7.53	0.32 ± 0.369	18	102
Control group	60	35 (58.33)	25 (41.67)	65.8 ± 7.10	0.65 ± 0.859	21	39
χ ^2^/t	/	0.139		0.696	2.788	6.400	
*P*	/	0.709		0.940	**0.003	*0.011	

Note: The number before the brackets in the table is an example, and the brackets are the proportion (%)

### Comparison of preoperative and postoperative D-dimer levels in patients with esophageal cancer

In the esophageal cancer group, the D-dimer concentration** was 1.21 ± 1.491 µg/mL on postoperativ-e day 1 significantly higher than that preoperatively (0.65 ± 0.859 µg/mL; F = 13.173, **P = 0.001). On postoperative day 14, the D-dimer concentration** (0.35 ± 0.254 µg/mL) was significantly lower t-han preoperative levels (0.65 ± 0.859 µg/mL; F = 9.427, **P = 0.003).

### Comparison of preoperative D-dimer levels in patients with different clinical characteristics in the esophageal cancer group

In the esophageal cancer group, the preoperative D-dimer level for well-differentiated tumors was 0.41 ± 0.331 µg/mL with a positivity rate of 21.05% (8/38). For moderately differentiated tumors, the D-dimer level was 0.45 ± 0.474 µg/mL with a positivity rate of 27.27% (12/44). In cases of poorly differ-entiated tumors, the D-dimer level was 1.14 ± 1.298 µg/mL with a positivity rate of 57.89% (22/38). A significant difference between the two groups was observed. The D-dimer level was 0.421 ± 0.367 µg/mL with a positivity rate of 18.75% (12/64) for postoperative pathological stages I–II and 0.92 ± 1.149 µg/mL, with a positivity rate of 53.57% (30/56) for stages III–IVA. There was a significant differe-nce between the two groups. Additionally, there were no significant differences in sex and age betwe-en participants in the two groups (all *P* > 0.05) (Table 2).

**Table 2:** Comparison of preoperative D-dimer levels in patients with different clinical characteristics (esophageal cancer group)

Project	*n*	D-dimer (µg/mL) ($\bar x$ ± s)	t-value	*P*-value	D-dimer (µg/mL)		χ^2^ value	*P*-value
					≤ 0.5 (*n*)	> 0.5 (*n*)		
Sex			-0.780	0.437			0.185	0.667
Male (n)	70	0.45 ± 0.489			46	24		
Female (n)	50	0.55 ± 0.896			32	18		
Age (years)			2.000	0.050			2.378	0.123
< 70	82	0.80 ± 1.00			48	34		
≥ 70	38	0.34 ± 0.203			30	8		
Tumor site			2.232	0.526			4.226	0.238
Upper chest	18	0.39 ± 0.194			12	6		
Middle chest	58	0.57 ± 0.498			36	22		
Lower chest	36	0.74 ± 1.269			28	8		
Esophagogastric junction cancer	8	1.47 ± 1.333			2	6		
Differentiation*			9.130	*0.010			6.580	*0.037
Well-differentiated	38	0.41 ± 0.331			30	8		
Moderately differentiated	44	0.45 ± 0.474			32	12		
Poorly differentiated	38	1.14 ± 1.298			16	22		
Postoperative pathological staging**			-2.330	*0.023			7.959	**0.005
I–II	64	0.42 ± 0.367			52	12		
III–IVa	56	0.92 ± 1.149			26	30		
T stage*			0.839	0.403			5.500	*0.019
T1–2	82	0.70 ± 0.949			59	23		
T3–4	38	0.56 ± 0.608			19	19		
N stage*			-0.656	0.513			5.018	*0.025
N0	68	0.61 ± 0.630			50	18		
N1–3	52	0.71 ± 1.087			28	24		

### Relationship between D-dimer level and local recurrence and distant metastasis of tumors

During the 3-year follow-up period, 12 patients were lost to follow-up, 36 died, 6 experienced anasto-motic recurrence, 12 had liver metastasis, 16 had lung metastasis, and 2 had bone metastasis. The aver-age D-dimer level* of these patients at the time of anastomotic recurrence or distant metastasis* was 1.48 ± 0.302 µg/mL, which was higher than the preoperative level of 0.51 ± 0.144 µg/mL, indicating a significant difference (F = 7.812, *P = 0.012).

### Factors affecting the 3-year survival rate after esophageal cancer surgery

**Fig. 1:**
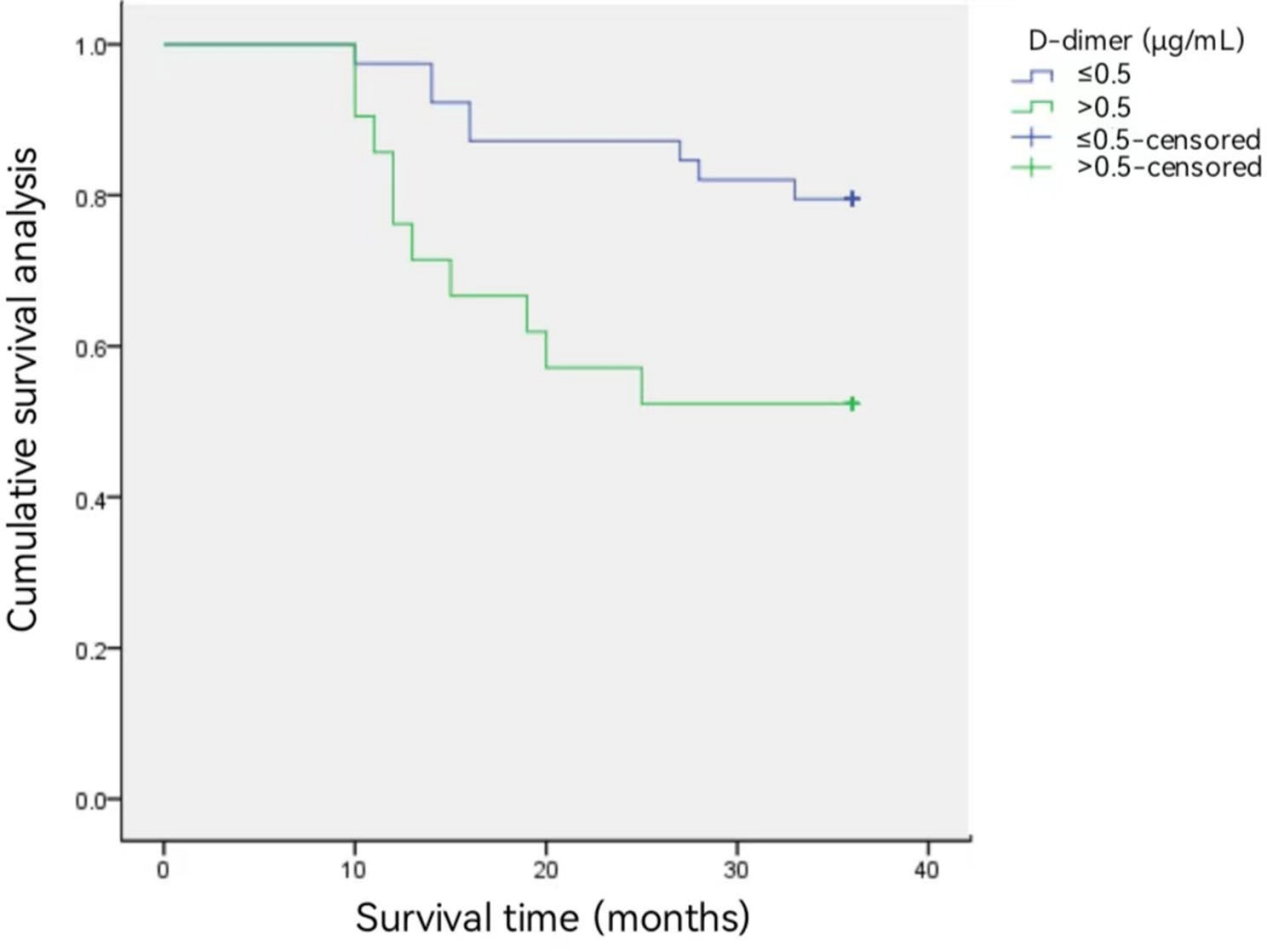
The 3-year survival rate in patients with esophageal cancer with D-dimer levels ≤ 0.5 µg/mL was 79.5% (62/78), which was significantly higher than the 52.4% rate (22/42) in patients with plasma D-dimer > 0.5 µg/mL (χ^2^ = 5.945, *P* = 0.015)

**Fig. 2:**
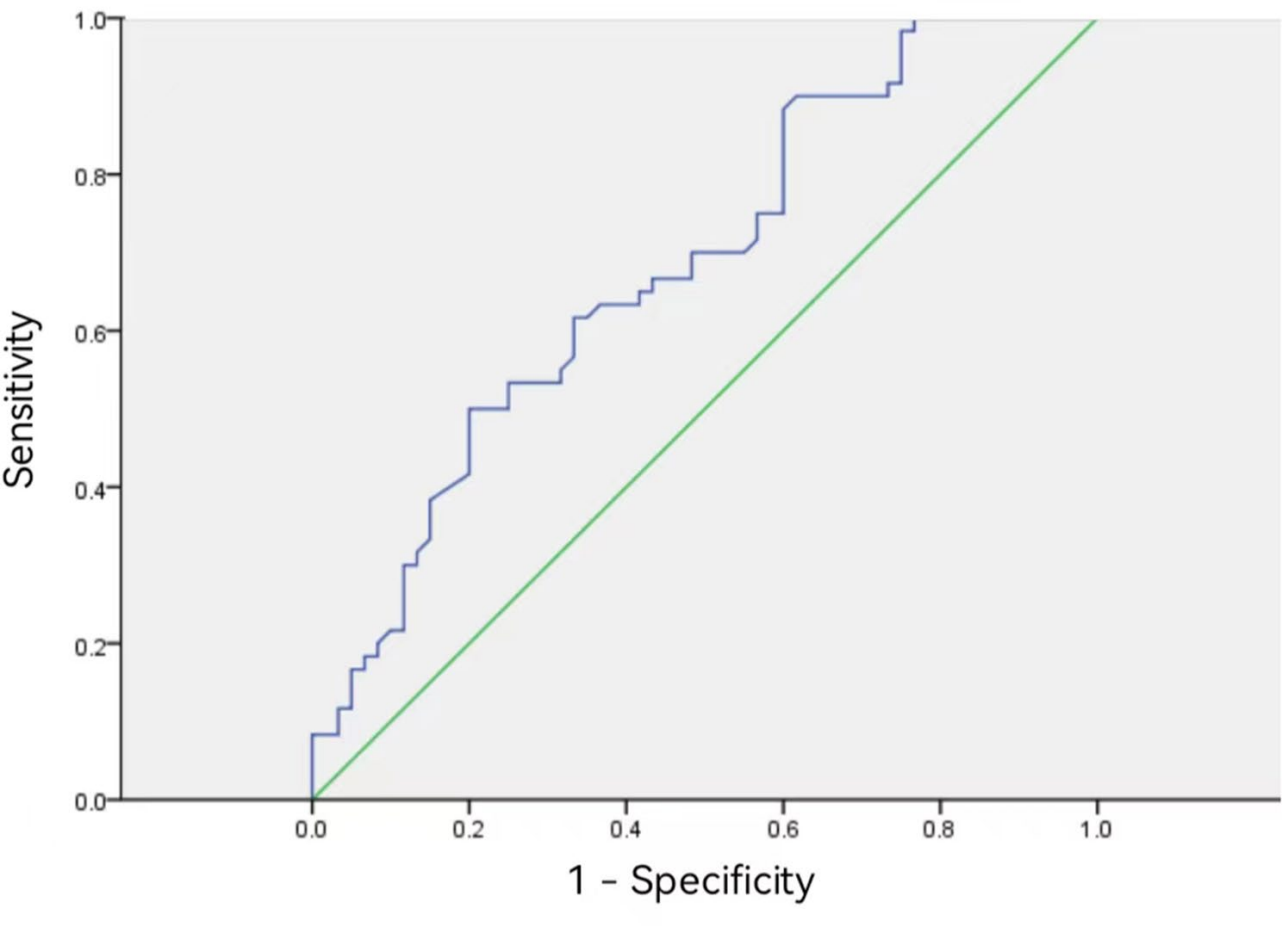
The 3-year survival rate in patients with esophageal cancer stratified by T stage and N stage. For subgroup analysis, the predictive value of the plasma D-dimer levels was significant in patients with T1–2 (*P* = 0.019, **A**), T3–4 (*P* < 0.001, **B**), N0 (*P* = 0.020, **C**), and N1–3 (*P* < 0.001, **D**)

**Fig. 3:**
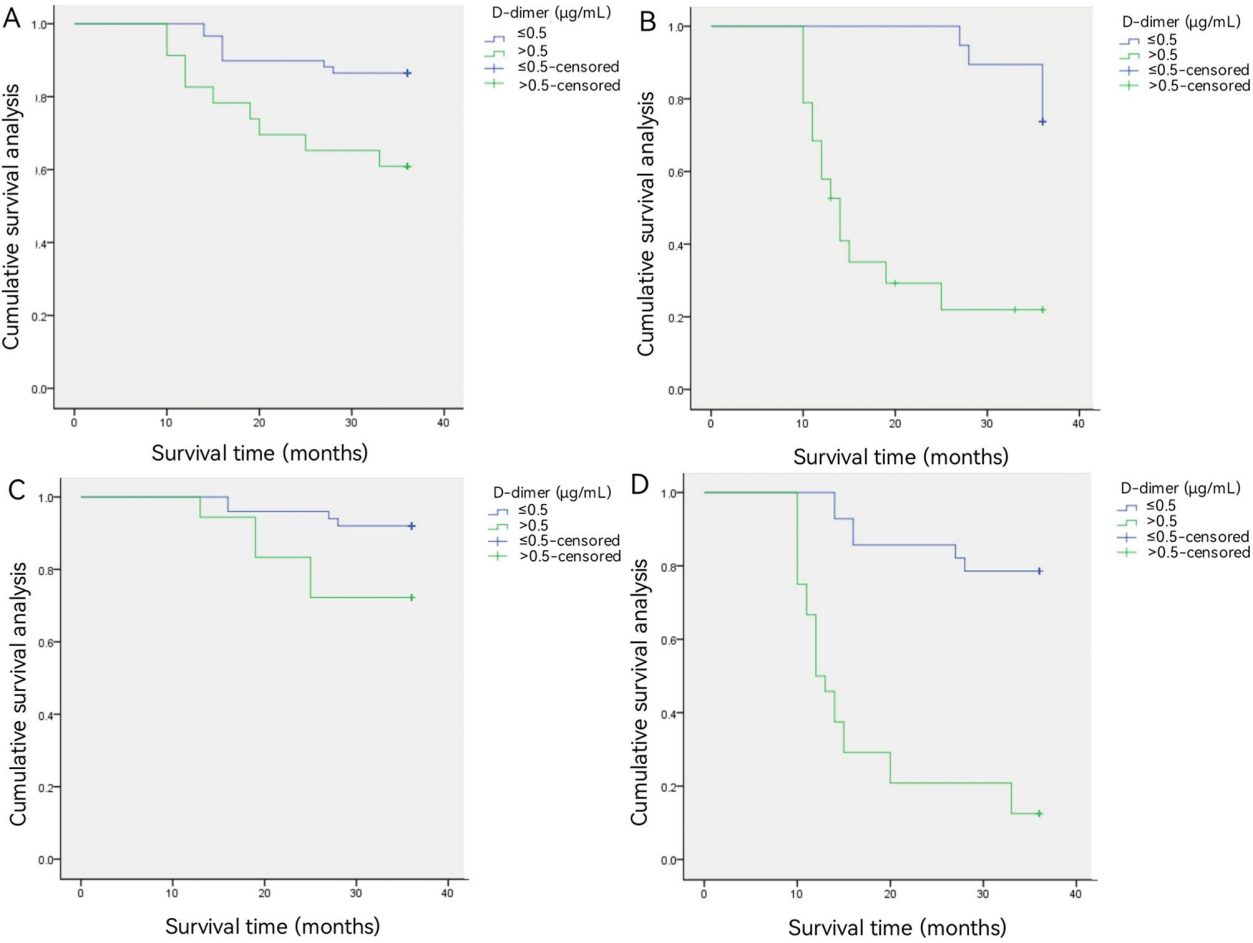
The area under the curve (AUC) value of the D-dimer level is 0.683, and D-dimer can predict the prognosis of esophageal cancer with a sensitivity of 88% and a specificity of 40%

The 3-year survival rate in patients with esophageal cancer with plasma D-dimer levels ≤ 0.5 µg/mL was 79.5% (62/78), which was higher than the 52.4% (22/42) in patients with plasma D-dimer > 0.5 µg/mL (Fig. 1). According to logistic multivariate analysis, tumor pathological stage* and preoperat-ive plasma D-dimer level* were independent prognostic factors affecting the 3-year survival rate in p-atients with esophageal cancer (both *P* < 0.05) (Table 3). We divided the patients into different subgr-oups according to T and N stages, and compared the 3-year survival rates of the two groups (all *P* < 0.05) (Fig. 2). The AUC value of the D-dimer level was 0.683. The D-dimer level could predict the pr-ognosis of esophageal cancer with a sensitivity of 88% and a specificity of 40% (Fig. 3).

**Table 3:** Multivariate logistic analysis results of the 3-year survival rate in patients with esophageal cancer

Relevant factor	B	SE	*P*-value	OR	95% CI
Tumor pathological staging*	-4.753	2.188	*0.030	0.009	0.000–0.629
Positive preoperative D-dimer levels*	-2.486	0.983	*0.011	0.083	0.012–0.572
Gender	0.940	0.721	0.193	2.559	0.623–10.518
Age	0.238	0.160	0.138	1.268	0.927–1.736
Tumor location	0.618	0.339	0.068	1.855	0.955–3.602
Tumor differentiation	0.225	0.296	0.448	1.252	0.701–2.236
Preoperative comorbidities	-0.006	0.017	0.709	0.994	0.960–1.028
Postoperative complications	0.825	0.457	0.071	2.282	0.932–5.589

## Discussion

Most patients with solid tumors have different degrees of abnormal coagulation function, including conditions such as hyperfibrinolysis, hemostatic dysfunction, abnormal coagulation function, or abnormal platelet activation [10]. In severe cases, this can lead to the formation of peripheral venous thrombosis [11], affecting clinical outcomes and prognoses in patients with cancer.

D-dimer—a molecular marker with specificity for fibrinolysis—is formed by the cleavage of cross-linked fibrinase, and its plasma concentration can directly reflect the coagulation state of the body. Therefore, D-dimer is widely used to diagnose disseminated intravascular coagulation, myocardial infarction, and thromboembolism [12]. 

Our study aimed to expand on this understanding by investigating the potential of D-dimer as a specific marker in patients with esophageal cancer. Our findings suggest that individuals with esophageal cancer exhibit a hypercoagulable state and heightened fibrinolysis, indicating the potential of D-dimer as a biomarker for this disease. Plasma D-dimer levels are a promising reference point for diagnosing esophageal cancer for several reasons. First, solid malignant tumors cause varying degrees of necrosis in surrounding healthy cells, releasing procoagulant substances into the bloodstream. This abnormal coagulation cascade leads to elevated levels of D-dimer in peripheral plasma [7]. Second, esophageal cancer is often diagnosed in the middle and late stages of progression. Patients experience malnutrition, emaciation, and cachexia, slowing peripheral venous blood flow and causing blood stasis. Third, tumor cells stimulate the release of procoagulant factors within the body, generating thrombin and accelerating fibrin degradation. This dynamic affects the overall coagulation function, pushing the body toward a hypercoagulable state [13]. Fourth, eliminating tumor cells triggers the release of procoagulant factors by natural killer cells, impacting coagulation processes [13]. Conversely, continuous tumor cell consumption, coupled with a decline in immune function, hinders the synthesis of blood coagulation factors and increases the risk of secondary hemorrhage [7]. 

In the esophageal cancer group, the D-dimer concentration on the first day after surgery was 1.21 ± 1.491 µg/mL, which was significantly higher than that before surgery (0.65 ± 0.859 µg/mL; F = 13.173, *P* = 0.001). Previous studies have suggested that patients are in a hypercoagulable state after surgery, which may be closely related to diffuse microthrombi in the surgical wound or surgical trauma. On postoperative day 14, the D-dimer concentration (0.35 ± 0.254 µg/mL) was significantly lower than the preoperative level (0.65 ± 0.859 µg/mL; F = 9.427, *P* = 0.003). It has been suggested that with the complete surgical resection of esophageal cancer and the patient’s recovery after surgery, the preoperative hypercoagulable state of patients with esophageal cancer can be improved. By dynamically monitoring perioperative D-dimer levels, we can detect a hypercoagulable state after esophageal cancer surgery; preventive anticoagulation may take up to 14 days after surgery time for reference.

During the 3-year follow-up period, 12 patients were lost to follow-up, 36 died, 6 experienced anasto-motic recurrence, 12 had liver metastasis, 16 had lung metastasis, and 2 had bone metastasis. The aver-age D-dimer level of these patients at the time of anastomotic recurrence or distant metastasis was 1.48 ± 0.302 µg/mL, which was higher than the preoperative level of 0.51 ± 0.144 µg/mL, indicating a s-ignificant difference (F = 7.812, *P = 0.012). In patients with stage III–IVA esophageal cancer, the pr-e-operative D-dimer level was 0.92 ± 1.149 µg/mL, significantly higher than that in patients with stag-e I–II of the disease. This escalation in D-dimer levels suggests a correlation between esophageal canc-er progression and increased hypercoagulability. This observation aligns with the work of Qifeng et a-l. [14], as tumor cells can disrupt blood vessel integrity during invasion, leading to hemorrhagic changes that activate the coagulation cascade and result in higher D-dimer levels [15]. Additionally, the hypercoa-gulable state of the blood promotes tumor cell mobility and metastasis, thereby accelerating the replic-ation of tumor cells [16]. Activating factors, such as the tissue factor present in patients’ blood, induce th-rombi formation, cell proliferation, and angiogenesis by affecting cell signaling pathways, facilitating the adhesion and proliferation of tumor cells [17]. In addition, the abnormal increase in D-dimer concent-ration can lead to blood stasis and provide a conducive environment for the hematogenous metastasis of tumor cells [18]. Consequently, a reciprocal cycle between tumor progression and the coagulation syst-em ensues, further emphasizing the potential significance of anticoagulant therapy in managing the ri-sk of metastasis and recurrence [19]. 

### Limitations

This study had some limitations. This was a single-center study with a relatively small sample size and short-term follow-up. Therefore, further validation of the results is necessary. Future steps involve expanding this research to other hospitals and conducting a statistical analysis of the 5-year postsurgical survival rate. The aim is to encompass a cohort of 60 patients with early esophageal cancer (cT1N0M0), measuring peripheral plasma D-dimer levels alongside tumor markers like carcinoembryonic antigen to establish a more comprehensive understanding of D-dimer’s diagnostic value.

## Conclusion

Our study highlights varying coagulation abnormalities in patients with esophageal cancer prior to surgery, characterized by significantly elevated peripheral plasma D-dimer concentrations. Although these levels normalized 14 days postoperatively, D-dimer levels increased again when the esophageal cancer recurred locally or metastasized. During the 3-year follow-up period, preoperative plasma D-dimer levels directly affected the 3-year survival rate in patients with esophageal cancer, underscoring its pivotal role in diagnosing esophageal cancer, monitoring, recurrent metastasis, and predicting patient prognosis.

## Data Availability

No datasets were generated or analysed during the current study.

## Abbreviations

AUC: area under the curve
